# Four-Dimensional Flow Magnetic Resonance Imaging for Assessment of Velocity Magnitudes and Flow Patterns in The Human Carotid Artery Bifurcation: Comparison with Computational Fluid Dynamics

**DOI:** 10.3390/diagnostics9040223

**Published:** 2019-12-13

**Authors:** Minh Tri Ngo, Chul In Kim, Jinmu Jung, Gyung Ho Chung, Dong Hwan Lee, Hyo Sung Kwak

**Affiliations:** 1 Department of Radiology and Research Institute of Clinical Medicine of Chonbuk National University, Biomedical Research Institute of Chonbuk National University Hospital, Jeon-ju 54907, Korea; ngominu@gmail.com (M.T.N.); chunggh@jbnu.ac.kr (G.H.C.); 2 Division of Mechanical Design Engineering, Chonbuk National University, Jeon-ju 54896, Korea; kci2850@jbnu.ac.kr (C.I.K.); jmjung@jbnu.ac.kr (J.J.); 3 Hemorheology Research Institute, Chonbuk National University, Jeon-ju 54896, Korea

**Keywords:** carotid artery, carotid bifurcation, flow patterns, computational fluid dynamics (CFD), four-dimension flow magnetic resonance imaging (4D flow MRI), velocity magnitudes

## Abstract

Purpose: Knowledge of the hemodynamics in the vascular system is important to understand and treat vascular pathology. The present study aimed to evaluate the hemodynamics in the human carotid artery bifurcation measured by four-dimensional (4D) flow magnetic resonance imaging (MRI) as compared to computational fluid dynamics (CFD). Methods: This protocol used MRI data of 12 healthy volunteers for the 3D vascular models and 4D flow MRI measurements for the boundary conditions in CFD simulation. We compared the velocities measured at the carotid bifurcation and the 3D velocity streamlines of the carotid arteries obtained by these two methods. Results: There was a good agreement for both maximum and minimum velocity values between the 2 methods for velocity magnitude at the bifurcation plane. However, on the 3D blood flow visualization, secondary flows, and recirculation regions are of poorer quality when visualized through the 4D flow MRI. Conclusion: 4D flow MRI and CFD show reasonable agreement in demonstrated velocity magnitudes at the carotid artery bifurcation. However, the visualization of blood flow at the recirculation regions and the assessment of secondary flow characteristics should be enhanced for the use of 4D flow MRI in clinical situations.

## 1. Introduction

Knowledge of the hemodynamic behaviors of blood flow in the vascular system is important in order to understand and treat vascular pathology [1,2,3]. While cardiovascular risk factors may decrease arterial distensibility and increase vessel wall thickness of the carotid artery [4], the development and progression of atherosclerosis in the carotid bifurcation is related to its local hemodynamic conditions, such as deceleration of blood flow, secondary flow, and shear stress—as has been shown in vitro, in animal models, and in magnetic resonance imaging (MRI) studies in vivo [3,5,6,7].

New medical imaging techniques that measure the flow in the circulation system can demonstrate the pathophysiology of atherosclerotic vascular disease [8]. Computational fluid dynamics (CFD) is an important method of visualizing blood flow and is widely used to evaluate the cardiovascular system’s local hemodynamic condition [9]. Recently, four-dimensional (4D) flow MRI has become a powerful tool for assessing blood flow. It is a feasible method of measuring the 3D distribution of the blood velocity vector field in vivo, allowing 3D flow structures to be examined with a single scan [10,11]. 4D flow MRI has been used to obtain data about the blood-flow characteristics of the cerebral vascular system, the aorta, as well as the carotid arteries, and can highlight local and global hemodynamic conditions [5,8,9,12,13,14].

The ability to effectively simulate the flow and pressure of the carotid artery is essential for researchers to model the performance of medical devices as well as predict the evolution of cardiovascular disease. Velocity measurements can be useful for quantification of different flow metrics through 4D flow MRI. But the spatial and temporal resolution of this technique is limited due to restricted scan-time in the clinical scenarios [15,16]. On the other hand, CFD is a computer simulation based on numerous assumptions requiring information about vascular morphologies, initial conditions, and boundary conditions. Previous studies showed that CFD can provide an accurate 3D velocity vector fields with high spatial and temporal resolution [17,18]. However, this method is rarely applied clinically due to the existing cumbersome process of model reconstruction and calculation.

There have been studies compared the measurement of 4D flow MRI with CFD in vivo. Steinman et al. [16] presented a method for quantifying wall shear stress and wall thickness at the human carotid bifurcation using a combination of MRI and CFD modeling and demonstrated this approach makes it ideal for carrying out future prospective studies of hemodynamics and plaque development or progression. Other reported similar findings that both 4D flow MRI and CFD have the potential to be used in the clinic and could provide vital information on disease progression [15]. Another study also compared the hemodynamics of intracranial aneurysm using 4D flow MRI and MR-based computational fluid dynamics and indicated a fair correlation between the two techniques [18]. However, one of the most challenging aspect of 4D flow MRI and CFD studies is having a large sample size due to existing technical limitations and a long time needed for the performances. On the other hand, difficulties in choosing appropriate boundary conditions have limited the application of this field in clinical practice. Hence, the aim of our study was to compare the results obtained through 4D flow MRI and image-based CFD techniques of the velocity and the flow pattern of 12 normal carotid artery bifurcations. With a larger sample size, we expect to provide more significant results. In this study, we generated 3D subject-specific models by employing flow results from 4D flow MRI for individual flow boundary conditions.

## 2. Material and Methods

### 2.1. Study Population

Our sample consisted of 12 healthy volunteers with no evidence of cardiovascular disease (8 men and 4 women). The mean age of the subjects was 30.4 ± 2 years (range, 28–35). Local institutional review boards approved this study protocol (Chonbuk National Hospital; the Ethics Committee of Chonbuk National University School of Medicine; CUH 2017-10-007, 1 October 2017).

### 2.2. Outline of the Workflow

**Figure 1:**
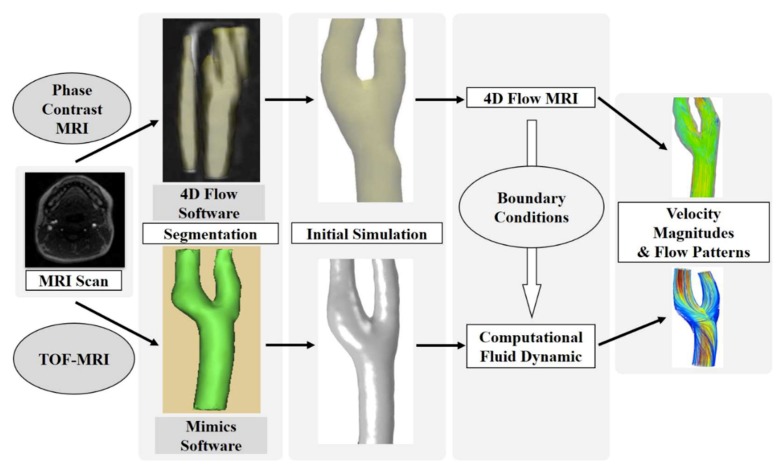
Flow chart of the hemodynamic velocity, and the flow pattern comparison process between four-dimensional (4D) flow magnetic resonance imaging (MRI) and computational fluid dynamics (CFD).

The study process is summarized in Figure 1, starting with the raw anatomical MR images. In each subject, a unilateral carotid artery was randomly selected. A 4D flow MRI subject-specific model was generated by the 4D flow software (4D flow v2.4.1nk, Siemens Healthcare, Erlangen, Germany). On the other hand, the CFD simulations were then performed by the segmentation of the MRI data and to achieve the most appropriate boundary conditions from the 4D flow MRI measurements. Both the 4D flow MRI and CFD were used to visualize blood flow in the carotid artery bifurcations of the 12 subjects. In addition, the velocity measured at the bifurcation plane was compared between the 4D flow MRI and CFD.

### 2.3. Magnetic Resonance (MR) Imaging Protocol

Examinations were performed on a 3T MRI (Magnetom Skyra, Siemens, Erlangen, Germany) using a combined 12-element head and a 6-element neck coil. Initially, the sequence time-of-flight (TOF) covering the carotid arteries was conducted to identify the exact location of the left and right carotid bifurcations. The scan parameters were as follows: ratio of repetition time (TR) to time to echo (TE) = 20/3.4 ms; flip angle = 20°; slice thickness = 0.8 mm; sensitivity encoding (SENSE) factor = 2.5; field of view = 200 × 200 mm; echo train length = 1; number of average (NEX) = 1. Image quality per artery was rated on a 4-point scale (1 = poor, 4 = excellent). Images with a quality <3 point were excluded from the study. The predominantly axial volume of 3D imaging was angulated to include the common carotid artery (CCA), the carotid bifurcation, the internal carotid artery (ICA), and the external carotid artery (ECA) based on the TOF-MRI data. The bifurcation in the left or right carotid arteries was positioned in the center of the 3D volume. The 4D flow MRI data were acquired with a non-contrast segmented 3D time-resolved RF-spoiled phase-contrast gradient-echo sequence with 3 directional velocity encoding. In combination with a k-space segmentation factor of 2 for each cardiac time frame, interleaved velocity encoding, i.e., consecutive measurement of the 1 reference and 3 velocity-sensitive scans, resulted in a temporal resolution (TR) = 47.5 milliseconds (msec) per time frame. The study parameters were as follows: dimensions were 176 × 176 × 52 millimeter (mm)^3^, spatial resolution was 1.1 × 1.1 × 1.1 mm^3^, number of frames were 14, and velocity sensitivity was 100 centimeter/second (cm/s) in all 3 directions. Data acquisition was prospectively gated to the cardiac cycle.

### 2.4. MR Imaging Data Processing

The resulting time-resolved anatomical and flow images were loaded into a software package (4D flow v2.4, Siemens Healthcare, Erlangen, Germany) for noise reduction, eddy-current correction, and calculation of 3D phase-contrast MRI as described previously [5,19].

Using the prototype 4D flow software, each subject’s 4D flow MRI simulation was generated allowing interactive visualization and evaluation of the 3D blood flow characteristics of the bifurcation in the left or right carotid artery. For 3D flow visualization, a 3D velocity streamline was used, which is based on the single time frame within the 4D flow MRI data. For the flow evaluation, retrospective quantification of the velocity blood flow can be performed for any arbitrary cross-sectional plane within the volumetric data by integrating the velocity inside the specified lumen.

### 2.5. Model Boundary Condition

**Figure 2:**
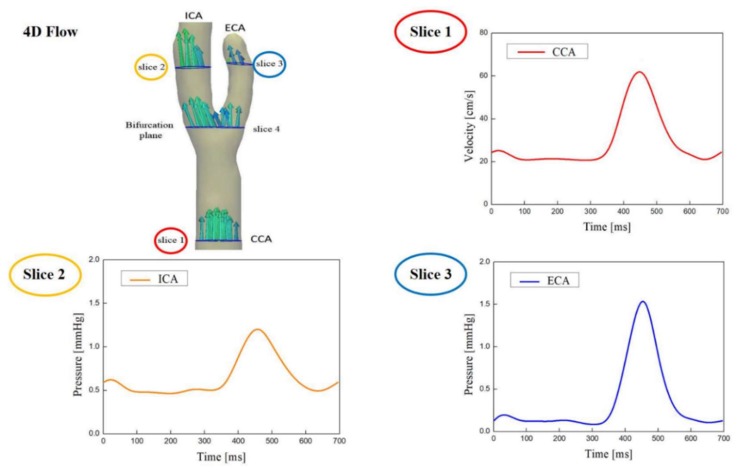
Time-varying axial indices of the carotid artery blood flow were analyzed using the 4D flow MRI at 4 slice locations along the vessel axial direction. Data from slices 1, 2, and 3 are used to determine the inflow boundary conditions and the outflow boundary conditions. Slice 4 (bifurcation plane) was used for velocity comparison with CFD results.

Following the MR geometry scan, time-varying axial indices of the carotid artery blood flow were analyzed using the 4D flow MRI at 4 slice locations along the vessel axial direction: Slices 1, 2, and 3 were measured at the CCA, ICA, and ECA; slice 4 was measured at the carotid bifurcation, as shown in Figure 2. Each plane was automatically angulated perpendicularly to the arterial lumen by the vessel navigation tool. Data from slices 1, 2, and 3 were used to determine the inflow and outflow boundary conditions. Slice 4 was used for velocity comparison with the CFD results.

### 2.6. MRI Reconstruction and 3D Carotid Artery Model

Twelve carotid artery geometries undergoing MRI scans were segmented to 3D geometry. The saved DICOM image files were converted from 2D images into 3D images using Mimics Software (version 20.0; Materialise NV, Leuven, Belgium). Inlets and outlets of all carotid artery geometries were cut uniformly in a plane perpendicular to the flow of blood. In the reconstructed geometry, unnecessary branches of the ECA were removed using an edit mask in a three-dimensional tool. All 3D carotid artery models were smoothed prior to the CFD simulation and analysis.

### 2.7. Computational Fluid Analysis

Multiphysics analysis of 12 carotid arteries was performed using COMSOL multiphysics 5.2 software (COMSOL Inc., Burlington, MA, USA). This study was performed with laminar flow analysis and blood was simulated with a density of 1066 kg/meter^3^ and a dynamic viscosity of 0.0035 pascal-seconds (Pa·s). The CFD simulation process of human carotid artery was as described previously [20]. The value of flow velocity obtained from the 4D flow MRI program was inputted to the inlet at the CCA, and the pressure values were inputted to the outlets at the ICA and ECA. These values were implemented in fouor cycles and computed as Womersley profiles (condition of full development). No-slip wall condition was given to the wall of the carotid artery model. The time-dependent analysis was applied to analyze the changes in the blood flow velocity and the velocity streamline in the carotid artery during four cardiac cycles.

### 2.8. Comparison of Velocity Magnitudes and Flow Pattern

The pair of corresponding axial slices on the 4D flow MRI simulation and CFD models were determined by using the junction point on the TOF-MRI as the reference point. The manual alignment was also used to ensure the match between the centroids of the two arterial cross-sections. This process was conducted manually for one slice uniformly at the carotid bifurcation to compare measurements obtained through the 4D flow MRI and CFD. For each analysis plane, the mean (averaged over the segmented lumen) was calculated. For the quantitative comparison, the maximum and minimum absolute blood flow velocities corresponding to the highest and lowest flow velocities through each cross-section during one cardiac cycle were used. In order to minimize the effect of initial transients, we used the result of the third cardiac cycle [21]. For comparison of the 4D flow MRI and CFD visualization of blood flow, we observe the flow pattern obtained by two methods. 3D velocity streamline was used to express the change of the blood flow in the carotid artery in both 4D flow MRI and CFD models. 3D images are displayed at peak systole to better illustrate complex blood flow patterns.

### 2.9. Statistical Analysis

For continuous variables, we calculated the mean ± standard deviation (SD). Data were analyzed using paired *t*-tests, with a *p*-value of 0.05 or less considered as statistically significant. A comparison of maximum and minimum blood flow velocities at the bifurcation plane by the 4D flow MRI and CFD was evaluated using the approach of Bland and Altman by calculating the mean (*d*) and SD of the difference. All analyses were performed using the SPSS statistical package, version 21.0 (SPSS Inc., Chicago, IL, USA).

## 3. Results

### 3.1. Correlation of Velocity Magnitude between the 4D Flow MRI and CFD Measurements

**Figure 3:**
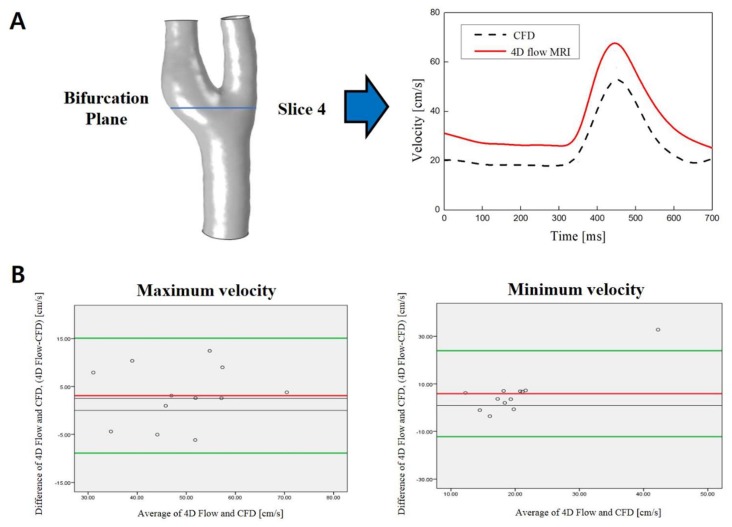
(**A**) Comparison of the velocity waveform at the bifurcation plane derived from 4D flow MRI (red line) and from CFD (dashed line); (**B**) Bland–Altman plot analysis of velocity data at the carotid bifurcation plane comparing the results of CFD and 4D flow MRI of maximum and minimum velocity. The green lines indicate the mean of the difference between 4D flow MRI and CFD ± 1.96 standard deviation; the red line shows mean difference value.

The blood flow velocities at the comparison plane for all 4D flow MRI simulations (*n* = 12) were compared to the CFD models. The velocity waveforms at the bifurcation plane for each subject provided by the 2 methods revealed a close similarity of the shape (Figure 3A). The mean maximum and minimum velocities of the 12 subjects measured by the 4D flow MRI and CFD were not significantly different (p ≥ 0.05), as shown in Table 1. While none of these results reached statistical significance, velocity measurements in 4D flow MRI were higher compared to CFD for both values at the carotid bifurcation plane (maximum velocity: 50.29 ± 11.66 centimeters/second (cm/s) on the 4D flow MRI vs 47.21 ± 11.02 cm/s on CFD; minimum velocity: 23.06 ± 11.84 cm/s on the 4D flow MRI vs 17.19 ± 3.85 cm/s on CFD). There was a 6.12% difference in maximum velocity and 25.46% difference in minimum velocity between the 4D flow MRI measurement and CFD calculation.

Bland–Altman plot analysis of velocity data at the carotid bifurcation plane comparing the results of the maximum and minimum velocities obtained in the 4D flow MRI and CFD is presented in Figure 3B. Most of the data points lie in the 95% limit band, showing good agreement between the 4D flow MRI and CFD measurements.

### 3.2. Comparison of Flow Pattern Derived by the 4D Flow MRI with CFD

**Figure 4:**
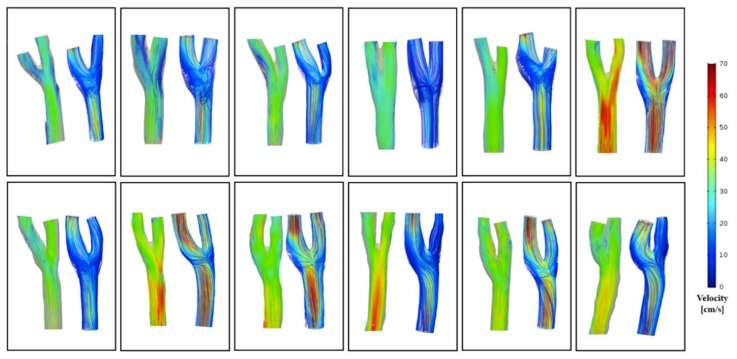
The flow patterns and velocity fields distribution for 12 carotid arteries at peak systole of 4D flow MRI (**left**) and CFD (**right**).

**Figure 5:**
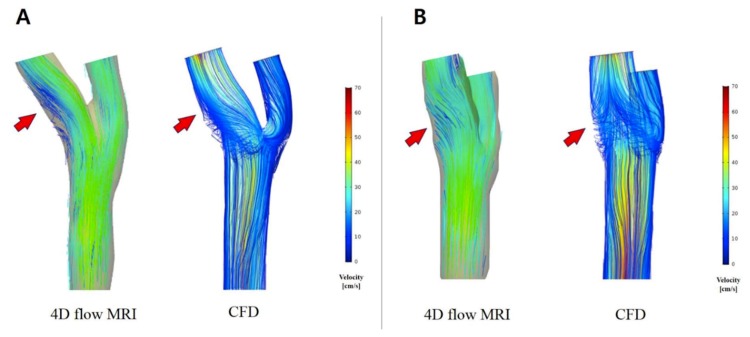
Visualization of velocity streamlines in the carotid artery bifurcation at peak systole using 4D flow MRI and CFD. 4D flow MR images of secondary flow direction and location (arrows) moderately coincide with the CFD visualization. (**A**) Lateral view; (**B**) posteroanterior view.

**Figure 6:**
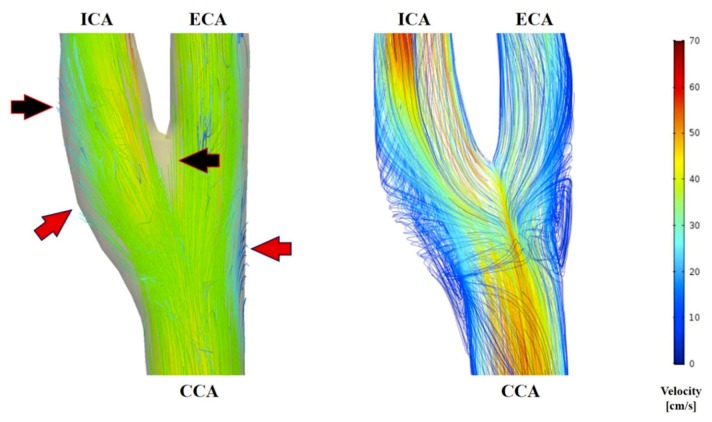
Visualization of velocity streamlines in the carotid artery at peak systole using 4D flow MRI (**left**) and CFD (**right**). In the blood flow patterns derived from 4D flow MRI, the velocity streamlines were disconnected at the segment of an arterial wall within the carotid bifurcation domain (black arrow) and recirculation regions were not well captured (red arrow).

Flow conditions for all subjects were analyzed with both the 4D flow MRI and CFD. Visual comparison between the 2 techniques of the 3D velocity streamline is provided in Figure 4. These visualizations show that the major flow structures and the secondary flow directions of the carotid arteries bifurcation observed in the 4D flow MRI moderately coincide with the CFD simulation (Figure 5). However, several differences were identified (Figure 6): (1) Secondary flows are of poorer quality when measured through the 4D flow MRI technique compared to CFD, making the flow pattern more parallel or laminar; (2) 3D velocity streamlines from the 4D flow MRI results stop suddenly at the segment of an arterial wall within the carotid bifurcation domain; (3) recirculation regions at the carotid sinus and carotid bifurcation, which is characteristic of transitional flow, were not well captured by the 4D flow MRI-performed flow patterns; (4) in the CFD models observed at the CCA and ICA, the maximum velocity magnitude of blood flow tended to be higher than in the 4D flow MRI simulation. By contrast, greater flow velocity in the ECA was measured by the 4D flow MRI simulation in almost all cases.

## 4. Discussion

The present study constructed 12 healthy subject-specific carotid artery bifurcation geometrical models using 4D flow MRI and CFD. Bland–Altman plot analysis of velocity data at the carotid bifurcation plane showed good agreement between 4D flow MRI and CFD measurements. In addition, the shape of the velocity waveform, which indicated values of low and high blood flow speeds corresponding to each phase of the cardiac cycle, showed good consensus between these two methods. While none of these results reached statistical significance, velocity measurements in 4D flow MRI were higher compared to CFD for both the maximum and minimum blood flow velocities at the carotid bifurcation plane. This is because the arterial blood flow patterns strongly depend on both the vessel geometry and flow conditions. The accuracy of anatomical models generated from MR images depends on the resolution of the image and the algorithms for reconstruction. In particular, the construction of anatomical models from the 4D flow MRI can lead to a smaller vessel caliber estimation compared to CFD, which in turn can results in higher velocities of the blood flow in 4D flow MRI measurement [22]. There was a 6.12% difference in maximum velocity and a 25.46% difference of minimum velocity calculations between the 4D flow MRI and CFD measurements in our study. The greater error of minimum velocity may be related to the high-velocity encoding (100 cm/s) used for data acquisition, which resulted in higher velocity noise in low velocities, and thus reduced the accuracy of the minimum velocity calculation obtained through the 4D flow MRI technique [23].

Several factors resulted in errors in measuring the 4D flow MRI velocity. A limited spatial resolution of 4D flow MRI results in partial volume effects at the arterial wall and a limited number of velocity data points within the blood flow from which to estimate the parameters of the model and positions of the wall. In addition, phase-contrast pulse sequence may introduce displacement artifacts since the effective encoding times for positions and velocity do not coincide [18,24]. Temporal resolution is an important factor as well. It is limited due to the trade-off between the temporal resolution and scan time in conventional 4D flow MRI sequences, and it has relatively low temporal resolution compared to CFD. This results in an average velocity though time, which reduces the ability to estimate transient events. To improve the accuracy of future MRI applications and to be able to measure the rapid variations in flow, it would be beneficial to increase spatial and temporal resolution [8,23]. Besides, the 4D flow MRI measurement sensitivity to low velocities values is governed by the setting selected for the velocity encoding (VENC) level. It is common for the VENC to be set sufficiently high to avoid aliasing in those regions with the highest velocity, and there is a velocity-to-noise ratio for those affected in slow flow regions, which are generally at the lumen boundary. Methods to increase the velocity-to-noise ratio, e.g., using multiple VENCs or reduced VENCs with phase unwrapping, would help to increase sensitivity to the conditions of low-flow [25,26].

The resolution, both spatial and temporal, is the main challenge to using 4D flow MRI. The primary effect of the limited spatial and temporal image resolution is the smoothing of the velocity pattern as a result of the average processes. Therefore, 4D flow MRI velocity patterns exhibit smaller secondary flow velocities compared to that of the CFD models, i.e., less swirling more parallel flows, and less transitional flows in the recirculation regions at the carotid sinus and bifurcation [22]. For clinicians, secondary flow patterns are of great importance in evaluating vascular disease [27]. Hence, mapping these behaviors with an acceptable resolution in 4D flow MRI is still not optimal.

The velocity fields from the 4D flow MRI and CFD showed large differences in the ECA velocity streamline maps. This may be due to differences in the geometry generated from the two methods in the axis CCA-ECA. The geometrical axis of CCA-ECA created by the 4D flow MRI segmentation appeared to be more vertical. As a result, the visualization of the ECA blood flow velocity magnitude in the 4D flow MRI simulation represented a greater value than in the CFD model in almost all cases.

The time it takes to calculate the CFD model depends on the computer system’s ability, the number of mesh, and the calculation conditions. The calculation time may vary from 5–8 h. Generally, 4D flow MRI measurements are acquired in 15–20 min for MR examination and in less than 1 h for processing. The 4D flow MRI was therefore thought to be a simple and easy instrument to measure and analyze carotid artery hemodynamics. As shown in this study, the results from the 4D flow MRI were not the same as those obtained by CFD, but in 3D flow patterns, there was a moderate to high degree of correlation. Our study found that the 4D flow MRI and CFD are complementary methods. Both techniques represent that the blood flow at the outer wall of the carotid sinus and carotid bifurcation is characterized by low flow, where the flow recirculation occurs. Despite the poorer resolution of the 4D flow MRI in the visualization of the recirculation regions, 4D flow MR images of secondary flow directions moderately coincided with the CFD visualization. This could provide additional insight into the pathophysiology and the prediction of the localization of high-risk atherosclerotic plaque at the carotid bifurcation.

Our study had several limitations. First of all, the approach of using 4D flow MRI as boundary conditions of CFD model introduces a bias for the subsequent comparison between the two techniques. However, the choice of the comparison plane at the carotid bifurcation, has some distances from the inlet and the outlet boundary conditions, may be helpful to reduce this bias. On the other hand, the purpose of this study was to merely assess the ability of the 3D specific model generation by the both techniques. With subject-specific measurements, our methods improve the feasibility in clinical practice. Other limitations were due to simplifications and uncertainties associated with our model. For example, blood viscosity can be associated with a variety of factors that researchers may not be available to identify with certainty. The rigidity of the wall assumed in this paper is another uncertainty associated with the simulation. Besides, the spatial and temporal resolutions used in the MRI scans are another limitation. With an acquisition time of 20 min in the conventional time-resolved 3D phase-contrast sequence, it was not possible to obtain better than 1.1-mm^3^ spatial resolution and 50-msec temporal resolution. A longer scan time would allow this resolution to be improved but it also increases the likelihood of motion artifacts due to patient movement, which can significantly degrade the accuracy of the results. Another limitation is the lack of evaluation of the 4D flow MRI technique’s interpatient reproducibility. A determination of this method’s statistical significance across multiple subjects will require a study with a larger number of patients. While the reproducibility of 4D flow MRI was high in healthy volunteers, further study is needed to determine whether it is also reliable for future applications in patients with carotid atherosclerotic disease. Recent advances in the acceleration technique for the 4D flow MRI sequence will lead to the widespread application of 4D flow MRI in clinical practice [28].

## 5. Conclusions

4D flow MRI and CFD show reasonable agreement in velocity magnitudes at the carotid artery bifurcation. The major flow structures and secondary flow directions of the carotid artery bifurcation observed in 4D flow MRI moderately coincided with CFD simulation. However, there were differences between the velocity fields between the 2 techniques. Despite these limitations, these techniques have the potential to aid in the clinical management of atherosclerotic disease. Future study should focus on approaches to overcome the limitations of 4D flow MRI in the visualization of blood flow at recirculation regions, as well as the assessment of secondary flow characteristics.

## Figures and Tables

**Table 1:** Blood velocities measured in 12 subjects at the bifurcation planes by four-dimensional (4D) flow magnetic resonance imaging (MRI) and computational fluid dynamics (CFD).

Velocity at the Carotid Bifurcation (*n* = 12)	4D Flow MRI	CFD	Difference	*p*
	Mean ± SD	Mean ± SD	Mean ± SD	
Maximum velocity (cm/s)	50.29 ± 11.66	47.21 ± 11.02	6.12%	0.05
Minimum velocity (cm/s)	23.06 ± 11.84	17.19 ± 3.85	25.46%	0.11

SD, standard deviation.
